# 2514 Disparities in care among patients presenting to the emergency department for nephrolithiasis

**DOI:** 10.1017/cts.2018.250

**Published:** 2018-11-21

**Authors:** Daniel Schoenfeld, Larkin Mohn, Ilir Agalliu, Joshua Stern

**Affiliations:** 1 Albert Einstein College of Medicine; 2 Columbia University Mailman School of Public Health; 3 Department of Epidemiology & Population Health, Albert Einstein College of Medicine; 4 Department of Urology, Albert Einstein College of Medicine and Montefiore Medical Center

## Abstract

OBJECTIVES/SPECIFIC AIMS: The prevalence of kidney stone disease has increased significantly in the United States in the last 2 decades. While several studies have reported that disparities in access to and quality of medical care exist, there is a need for a more thorough investigation of factors that negatively impact patients seeking care specifically for kidney stone disease. We sought to examine whether kidney stone patients received different standard of care in the emergency department (ED) according to their race/ethnicity, gender, age, body mass index, socioeconomic status (SES), and insurance status. METHODS/STUDY POPULATION: We conducted a retrospective study of patients presenting to the ED at Montefiore Medical Center between January 1, 2014 and December 31, 2016. Patients with a diagnosis of nephrolithiasis were identified using ICD-9/10 codes and electronic chart review was used to assess each patient’s ED course as well as to gather sociodemographic information. The primary outcomes of interest were administration of pain medication, prescription of alpha-1 antagonists to facilitate stone passage and whether or not patients received CT scan or ultrasound. Associations of these outcomes with age categories, sex, race/ethnicity, body mass index category, SES and insurance status were examined using multivariate logistic regression models. RESULTS/ANTICIPATED RESULTS: A total of 1200 patients were included in this analysis of which 616 (51%) were women. A large proportion of patients were minorities (40% Hispanic and 15% non-Hispanic African-American), whereas 21% were Caucasian and 24% declined to report race/ethnicity. Patients between the ages of 55–64 and those older than 65 were less likely to receive pain medication compared to younger patients aged <35 years (OR=0.48, 95% CI: 0.27–0.86 and OR=0.46, 95% CI: 0.21–1.00, respectively). Women were less likely than men to undergo any form of diagnostic imaging (OR=0.52, 95% CI: 0.35–0.76) including CT scan (OR=0.50, 95% CI: 0.35–0.72). Similarly, patients in the lowest quintile of SES received less imaging than patients in higher SES categories (OR=0.50; 95% CI: 0.27–0.90). Furthermore, African Americans (both genders) and women were less likely to be prescribed an alpha antagonist medication (e.g., tamsulosin) to facilitate stone passage compared with White patients (OR=0.61, 95% CI 0.36–1.03) and men (OR=0.68, 95% CI: 0.49–0.92), respectively. DISCUSSION/SIGNIFICANCE OF IMPACT: We found that multiple disparities exist among patients presenting to the ED for nephrolithiasis. A more thorough investigation into the causes of these disparities is warranted to limit their impact on patient care.

